# Draft Genome Sequence of Pseudomonas carnis Strain 23-145, Causing Blue Discolorations on Rabbit Carcasses

**DOI:** 10.1128/mra.00136-23

**Published:** 2023-04-05

**Authors:** Michael Biggel, Julia Lienhard, Roger Stephan

**Affiliations:** a Institute for Food Safety and Hygiene, Vetsuisse Faculty, University of Zürich, Zürich, Switzerland; b Section of Veterinary Bacteriology, Institute for Food Safety and Hygiene, Vetsuisse Faculty, University of Zürich, Zürich, Switzerland; DOE Joint Genome Institute

## Abstract

Here, we report the genome sequence of Pseudomonas carnis strain 23-145, which was recovered from a rabbit carcass with blue discolorations. The strain harbored two *trpABCDF* loci involved in tryptophan biosynthesis, which is characteristic of blue-pigment-producing Pseudomonas strains.

## ANNOUNCEMENT

**FIG 1:**
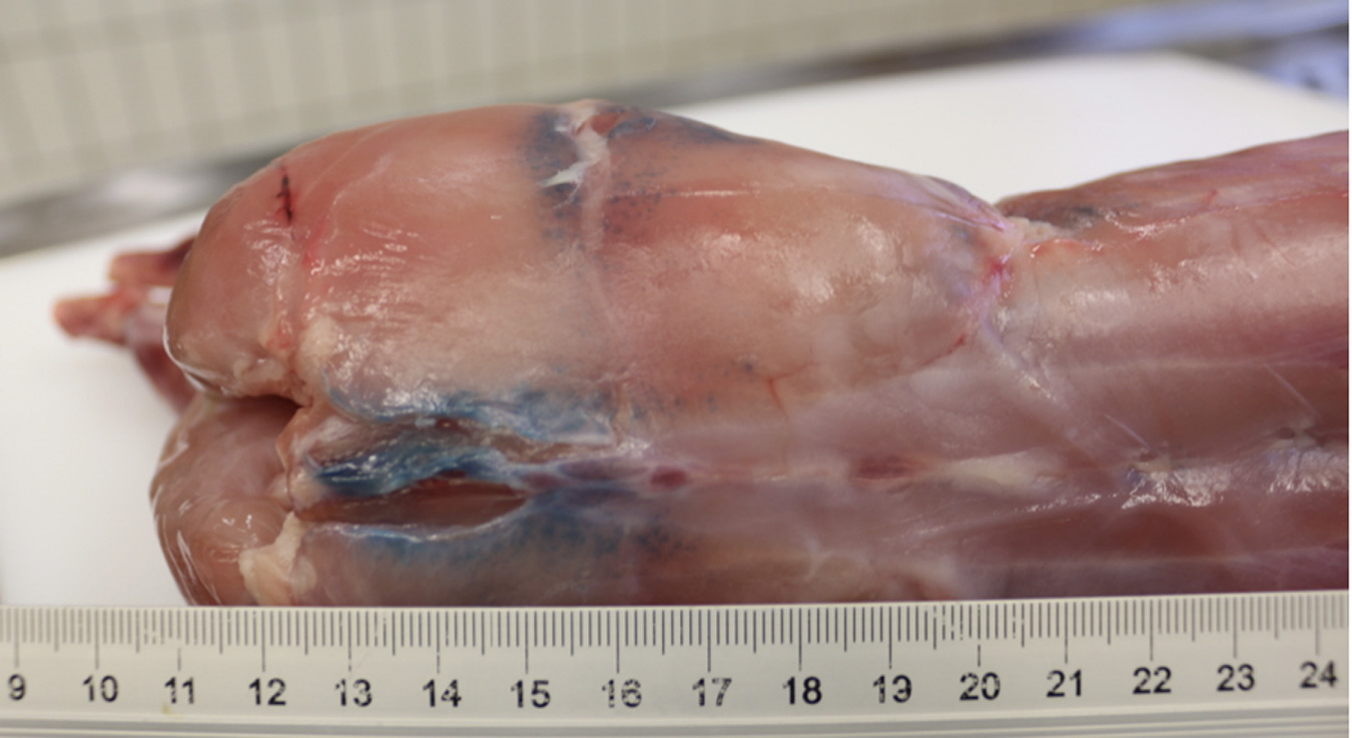
Blue discolorations on a rabbit carcass 10 days after slaughter.

Some species of the Pseudomonas fluorenscens group can spoil food by producing pigments that lead to color changes (1–3). The aim of this work was to identify the microbiological cause of blue discolorations found on the carcass of a fattening rabbit 10 days after slaughter in an abattoir in Switzerland in January 2023 (Fig. 1). For the microbiological examination, the surface of the discolored meat was disinfected with ethanol. Swab samples from cut meat were then spread on various agar plates (Columbia blood agar, Gassner agar, Schaedler agar, and Columbia nalidixic acid agar [CNA]; Thermo Fisher Scientific). The plates were incubated at 37°C aerobically and anaerobically for up to 2 days. Matrix-assisted laser desorption ionization–time of flight mass spectrometry (MALDI-TOF MS) (Bruker Daltronics) was used for preliminary species identification of grown colonies. A low-grade mixed flora consisting of Carnobacterium divergens, Staphylococcus saprophyticus, and two morphologically distinct Pseudomonas species was found. MALDI-TOF MS typed one Pseudomonas sp. isolate (23-145) as a representative of the Pseudomonas fluorescens group and the second as either Pseudomonas lundensis or Pseudomonas taetrolens.

To determine which of the two Pseudomonas isolates was responsible for the observed color changes, we spread the isolates on distinct surfaces of a fresh rabbit carcass and stored the carcass at 4°C. After 4 days, the surface area inoculated with isolate 23-145 showed an incipient color change, which increased massively in the following days. The area inoculated with the second isolate (Pseudomonas lundensis or Pseudomonas taetrolens) did not show visible changes over the same period.

Genomic DNA of isolate 23-145 was extracted using the DNeasy blood and tissue kit (Qiagen) from subcultures obtained from single colonies that had been grown for 24 h at 37°C on sheep blood agar. Libraries were prepared using the Nextera DNA Flex library preparation kit (Illumina) and sequenced on the Illumina MiniSeq platform (2 × 150 bp). Read trimming and quality control were performed with fastp v0.23.2 (4). A draft assembly was generated from 347 Mbp of read data (2,332,296 reads [coverage, 52×]) using SPAdes v3.14.1 (5) implemented in Shovill v1.1.0 (https://github.com/tseemann/shovill). The genome was annotated using the NCBI Prokaryotic Genome Annotation Pipeline (PGAP) v6.4 (6). *In silico* taxonomic classification was performed using ribosomal multilocus sequence typing (rMLST) (7) (https://pubmlst.org). Default parameters were used for all software unless otherwise stated.

Isolate 23-145 was identified by rMLST as Pseudomonas carnis, a member of the P. fluorenscens group. The 6.3-Mbp draft assembly consisted of 186 contigs (*N*_50_, 81.6 kb; GC content, 59.95%) and contained two sets of the tryptophan biosynthesis genes *trpABCDF*. The presence of multiple *trpABCDF* homologues has recently been linked to pigment (indigo derivate) production and discoloration of food products (8, 9). Our data provide further evidence that the accessory tryptophan biosynthesis genes could be used as diagnostic targets for the identification of pigment-producing Pseudomonas strains.

### Data availability.

The draft assembly is available at NCBI GenBank under assembly accession number GCA_028863905.1. The BioProject and SRA accession numbers are PRJNA935533 and SRR23495213, respectively.
